# The effect of 5-alpha reductase inhibitors on the detection of prostate cancer with multiparametric magnetic resonance imaging and prostate biopsy

**DOI:** 10.3325/cmj.2024.65.417

**Published:** 2024-10

**Authors:** Mefail Aksu, Ural Oğuz, Serdar Aslan, Erhan Demirelli, Özay Demiray, Birgül Tok, Doğan Sabri Tok, Ercan Öğreden

**Affiliations:** 1 Department of Urology, Giresun University School of Medicine, Giresun, Turkey; 2 Department of Radiology, Giresun University School of Medicine, Giresun, Turkey; 3 Department of Urology, Ministry of Health, Giresun Training and Research Hospital, Giresun, Turkey; 4 Department of Pathology, Giresun University School of Medicine, Giresun, Turkey

## Abstract

**Aim:**

To evaluate the effect of 5 alpha-reductase inhibitor (5-ARI) treatment on prostate cancer detection by multiparametric magnetic resonance imaging (mpMRI).

**Methods:**

We retrospectively collected data on 429 patients who underwent mpMRI before prostate biopsy in the Urology Department of Giresun University Training and Research Hospital between March 2018 and December 2021. The patients were categorized as those who had never been treated with 5-ARI (n = 359) and those who were treated with 5-ARI for more than six months (n = 70). The two groups were compared in terms of age, mpMRI findings, and pathology results.

**Results:**

The number of patients with Prostate Imaging-Reporting and Data System (PIRADS) 3 score was significantly higher in the 5-ARI group (37.1% vs 20.6%; *P* = 0.009). The groups did not differ in terms of malignant pathological findings (48.5% in the non-5-ARI vs 47.1% in the 5-ARI group; *P* = 0.505). The detection rates of malignant pathological findings in PIRADS 3 and PIRADS 5 patients were similar between the 5-ARI and non-5-ARI group. However, malignancy detection rate in PIRADS 4 patients was significantly higher in the non-5-ARI group (*P* = 0.031). In the non-5-ARI group, the sensitivity was 56.4% and the specificity was 79.7%. In the 5-ARI group, the sensitivity was 84.9% and the specificity was 56.8%.

**Conclusion:**

In patients with suspected prostate cancer, 5-ARI intake may alter lesion mpMRI characteristics and PIRADS distribution on mpMRI. 5-ARI intake should be reported to the radiologist.

Prostate cancer is the most common type of cancer in male patients, ranking second (behind lung cancer) in terms of cancer-related mortality. The lifetime risk of prostate cancer is one in nine (1). Due to the high incidence of prostate cancer, appropriate diagnostics, treatment, and follow-up are important. Prostate cancer is suspected based on the results of prostate-specific antigen (PSA) test and digital rectal examination, and the definitive diagnosis is made by prostate biopsy (2). In addition, magnetic resonance imaging (MRI) has been used since the 1980s as a non-invasive imaging method in the diagnosis and staging of the disease, monitoring its local spread, and the detection of lymph node metastases (3,4). Multiparametric magnetic resonance imaging (mpMRI) encompasses several different MRI techniques, such as T2 weighted (T2W) imaging for anatomical evaluation, diffusion-weighted imaging (DWI) for functional evaluation, dynamic contrast imaging (DCI), and MR proton spectroscopy (3).

5-alpha reductase inhibitors (5-ARI) are a first-line treatment widely used for benign prostatic hyperplasia (BPH) (5-7). More than half of men affected by BPH develop symptomatic disease between the ages of 50 and 70 (8), which is the recommended age for early detection of prostate cancer (9). 5-ARI treatment after three to six months reduces the need for surgery by approximately 50% and prostate volume by 25% (10,11). It also reduces serum PSA levels by 50% after six months (12).

Some studies have shown that 5-ARI reduces the detection of prostate cancer and alters PIRADS scores of lesions visible on mpMRI (13-15). However, other studies investigating the effects of 5-ARI on MRI detection of prostate cancer have yielded conflicting results. In light of these findings, we aimed to evaluate how prostate cancer is affected radiologically and pathologically by the use of 5-ARI.

## Patients and methods

**Figure 1:**
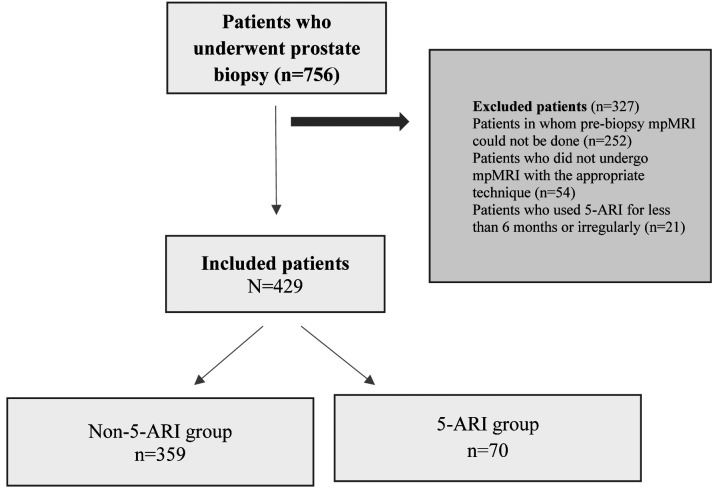
Study flowchart. 5-ARI – 5 alpha-reductase inhibitors; mpMRI –multiparametric magnetic resonance imaging.

In this retrospective study, we reviewed the medical records of 756 patients who underwent transrectal ultrasound cognitive prostate biopsy in the Urology Department of Giresun University Training and Research Hospital between March 2018 and December 2021. The study enrolled patients who had undergone mpMR imaging, including DWI, T2W, and DCI sequences, before biopsy. Patients taking 5-ARI for less than 6 months or irregularly were excluded. The final sample involving 429 patients (Table 1) was categorized into those who had never taken 5-ARI (n = 359) and those who were taking 5-ARI for more than 6 months (n = 70) (Figure 1). We gathered data on age, PSA, PSA density (PSAD), prostate volume, mpMRI findings, and pathology results. The study was approved by the Clinical Research Ethics Committee of the Ministry of Health Kanuni Training and Research Hospital.

**Table 1:** Prostate Imaging-Reporting and Data System (PIRADS) distributions and pathological results in the group not using 5 alpha-reductase inhibitors (5-ARI) and the group using 5-ARI

		Group						χ^2^ test	
		non-5-ARI		5-ARI		total			
		n	%	n	%	n	%	χ^2^	p
PIRADS	3	74	20.6	26	37.1	100	23.3	9.496	0.009
	4	150	41.8	26	37.1	176	41.0		
	5	135	37.6	18	25.7	153	35.7		
	total	359	100.0	70	100.0	429	100.0		
Pathology	benign	185	51.5	37	52.9	222	51.7	1.365	0.505
	malignant	174	48.5	33	47.1	207	48.3		
	total	359	100.0	70	100.0	429	100.0		

Total serum PSA levels were determined with an electrochemiluminescence immunoassay using the cobas® 8000 modular analyzer, series e-602 module (Roche Diagnostics, Indianapolis, IN, USA). We gathered data on measured PSA rather than on corrected PSA because of different periods of 5-ARI usage. PSAD was calculated by dividing PSA by prostate volume, obtained from MR images.

mpMR images (DWI, T2W, DCI) were evaluated by a radiologist with 10 years of uroradiology experience. mpMRI findings were graded according to the Prostate Imaging-Reporting and Data System (PIRADS), v. 2 (3), evaluation categories. Laboratory findings and pathology reports were evaluated according to the ISUP grading system (16).

### Statistical analysis

The normality of distribution of continuous variables was tested with a Kolmogorov-Smirnov test. Variables are presented as means and standard deviations, or medians and interquartile ranges (IQR). Categorical variables were compared with a χ^2^ test, and continuous variables with a Mann-Whitney U test. We used receiver-operating characteristic (ROC) curves to assess the diagnostic performance of mpMRI in detecting prostate cancer. Sensitivity and specificity were calculated. *P* values below 0.05 were considered significant. Statistical analysis was carried out with SPSS, version 22.0 (IBM Corp., Armonk, NY, USA).

## Results

The mean age did not significantly differ between the groups (66.57 in the non-ARI group vs 68.66 in the ARI group; *P* = 0.055). The median PSA and median PSAD were significantly higher in the non-ARI group (7.12 ng/mL vs 5.37 ng/mL, *P* = 0.001; 0.14 vs 0.10; *P* = 0.006, respectively). There was no difference in prostate volume (non-ARI group: 60.2 cm^3^ vs 59.9 cm^3^ in the ARI group; *P* = 0.835)

**Figure 2:**
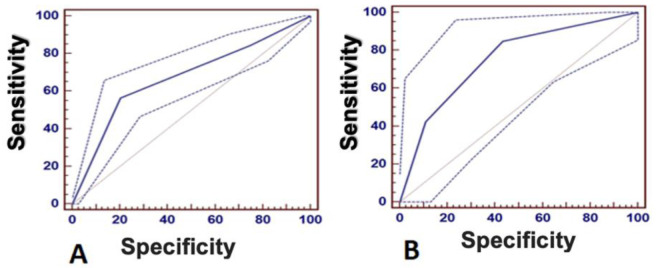
Receiver operating characteristic curve of multiparametric magnetic resonance imaging in (**A**) the group not using 5 alpha-reductase inhibitors (area under the curve: 0.673; 95% confidence interval 0.621-0.721); (**B**) the group using 5 alpha-reductase inhibitors (area under the curve: 0.754; 95% confidence interval 0.636-0.849).

In the non-5-ARI group, the sensitivity was 56.4%, and the specificity was 79.7%. In the 5-ARI group, the sensitivity was 84.9%, and the specificity was 56.8%. Areas under the curve (AUC) in the non-5-ARI group and ARI group were 0.673 (95% CI 0.621-0.721) and 0.754 (95% CI 0.636-0.849), respectively (Figure 2).

When MRI results were compared, in the non-5-ARI group, PIRADS 3 was found in 74 patients (20.6%), PIRADS 4 in 150 patients (41.8%), and PIRADS 5 in 135 patients (37.6%). In the 5-ARI group, PIRADS 3 was found in 26 patients (37.1%), PIRADS 4 in 26 patients (37.1%), and PIRADS 5 in 18 patients (25.7%). The number of patients with PIRADS 3 was significantly higher in the 5-ARI group than in the non-5-ARI group (37.1% vs 20.6%; *P* = 0.009) (Table 1).

**Figure 3:**
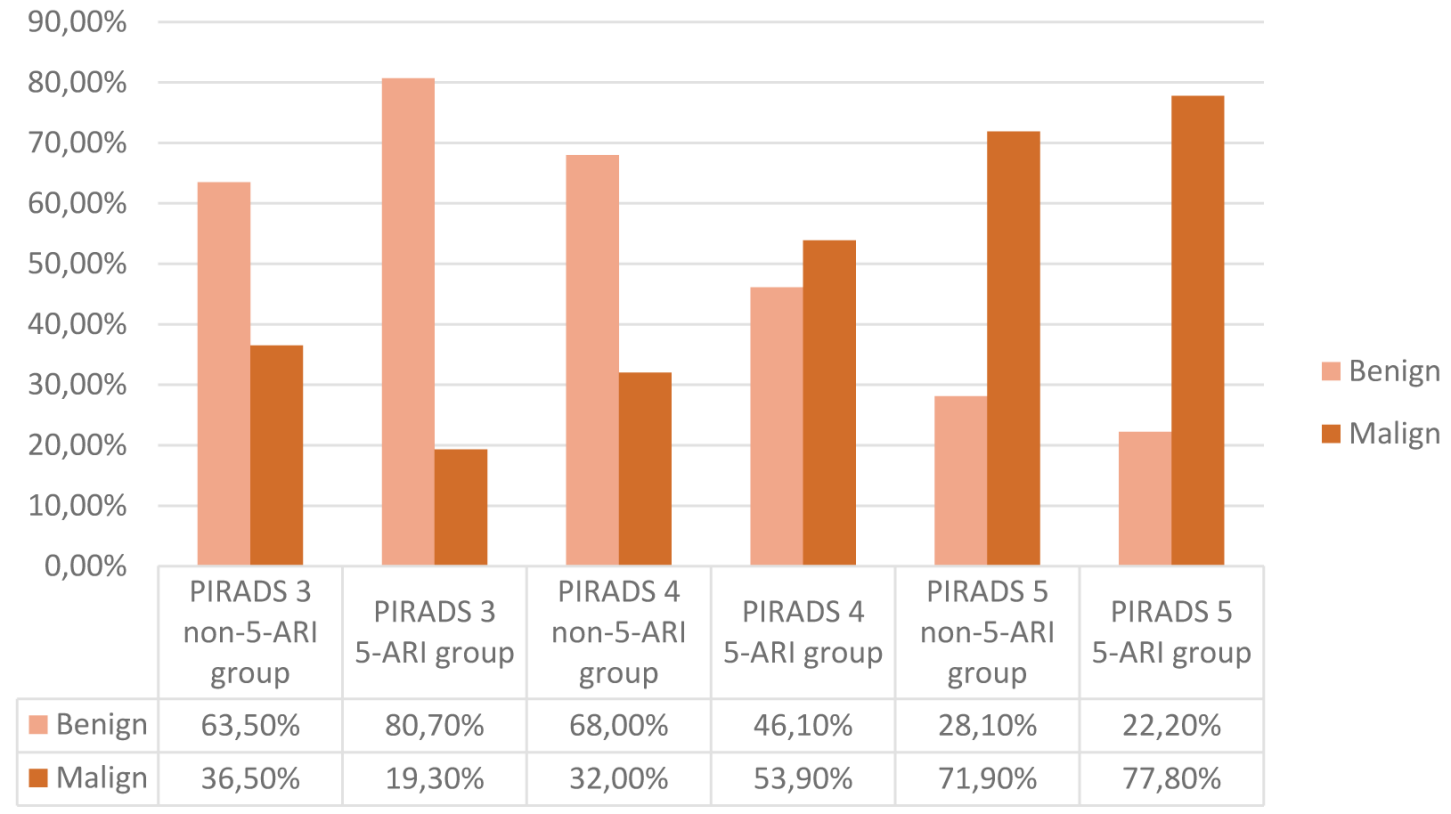
Prostate Imaging-Reporting and Data System (PIRADS) subgroup malignancy rates in the group not using 5 alpha-reductase inhibitors (5-ARI) and the group using 5-ARI.

The malignancy detection rate in patients with PIRADS 3 score was 36.5% in the 5-ARI group and 19.3% in the non-5-ARI group (*P* = 0.105). The malignancy detection rates in patients with PIRADS 4 (32% vs 53.9%) and PIRADS 5 (71.9% vs 77.8%) were higher in the 5-ARI group, but the difference was significant only for the PIRADS 4 group (*P* = 0.031 and *P* = 0.597, respectively) (Figure 3). There was no significant difference in total malignant pathological findings between the groups (48.5% in the non-ARI group, 47.1% in the ARI group; *P* = 0.505) (Table 1).

## Discussion

This study showed that in patients with suspected prostate cancer, 5-ARI intake may alter lesion MRI characteristics and PIRADS distribution on mpMRI. The number of patients with PIRADS 3 lesions was higher in the 5-ARI group. In both groups, the cancer detection rate in patients with PIRADS 3 lesions was lower than in patients with PIRADS 4 and PIRADS 5 lesions.

In some studies, the use of 5-ARI affected the detection of prostate cancer and the interpretation of mpMRI (13-15). For example, Andriole et al reported that, compared with placebo, 5-ARI reduced the risk of prostate cancer by lowering the level of dihydrotestosterone, but the detected malignancies were of higher grade (13). Iczkowski et al reported that 5-ARI affected cellular involution and epithelial shrinkage of the prostate tissue, and increased the stromal/epithelial ratio in prostate cancer (17). 5-ARI can induce significant phenotypic changes in both benign and cancerous prostate tissue. In the MAPPED study (14), patients treated with 5-ARI experienced a reduction in prostate cancer volume on T2WI compared with placebo-treated patients (*P* < 0.0001). In this context, the intake of 5-ARI is expected to affect the interpretation of mpMRI (18,19).

However, other studies investigating the effects of 5-ARI on the quantitative parameters of cancerous and benign prostate lesions have yielded conflicting results. Giganti et al found that 0.5 mg 5-ARI, administered daily for six months, did not significantly affect T_2_ contrast or T_2_ relaxation values in men under active surveillance for prostate cancer (18). In a retrospective study involving 20 patients, Starobinets et al found a lower discrimination ability of T2WI between prostate cancer and benign tissue in the 5-ARI group than in controls, but for functional MR measurements, the discrimination was higher in the 5-ARI group (19).

In the study by Bekci et al, the sensitivity of mpMRI for detecting prostate cancer was 96%, and the specificity was 43% (20). At the same time, Loggitsi et al reported the sensitivity and specificity of mpMRI for prostate cancer to be 53% and 90.3%, respectively (21). In a meta-analysis of 3857 patients, the sensitivity of PIRADS v. 2-guided mpMRI to detect prostate cancer was 89% and the specificity was 73% (22). In our study, the sensitivity in the non-5-ARI group was 56.4%, and the specificity was 79.7%. In the 5-ARI group, the sensitivity was 84.9%, and the specificity was 56.8%. The sensitivity and specificity values in the non-5-ARI group were similar to those reported in previous studies. However, the sensitivity in the 5-ARI group was higher.

In our study, AUC analysis showed that the predictive ability of mpMRI to detect prostate cancer in the 5-ARI group was higher than in the non-5-ARI group. 5-ARI may cause more pronounced radiological changes in non-malignant prostate tissues; accordingly, mpMRI may have greater sensitivity in the detection of the cancerous area.

Giganti et al (15) showed that 5-ARI treatment increased tumor apparent diffusion coefficient and reduced conspicuity on DWI. The authors concluded that the prostate biopsy threshold in mpMRI should be lower in patients who used 5-ARI than in the general population (15). In our study, the number of patients with PIRADS 3 lesions was significantly higher in the group using 5-ARI. In addition, although the result was not significant, PIRADS≥4 lesions were less frequently observed in the 5-ARI group. This suggests that the use of 5-ARI may decrease the contrast enhancement of the lesions and DWI sequence visibility.

Other studies found similar PIRADS score distributions as in our study (23,24). In a study designed similarly to ours, Kim et al found a significant difference in the prostate cancer detection rate between the 5-ARI and non-5-ARI groups only in the PIRADS category ≥4 (75.0% vs 53.7%, *P* = 0.031) (25). Wang et al (26) observed a comparable distribution of PIRADS categories between the 5-ARI and non-5-ARI group. However, the overall prostate cancer detection was significantly reduced in the 5-ARI group (68.0% vs 46.3%) (26). The recently reported Prostate MRI Outcomes Database study concluded that 5-ARI exposure did not affect the PIRADS distribution (27). In this study, a higher rate of high-grade prostate cancer (ISUP>3) was observed in 5-ARI users with PIRADS 5 compared with 5-ARI non-users.

The total malignancy rates of both groups were similar. Although patients with PIRADS 3 and 5 scores from both groups had similar malignancy rates, 5-ARI patients with PIRADS 4 had a significantly higher malignancy rate. This finding should be taken into consideration in clinical practice.

Our study has some limitations. Although there is no prospective study about this topic in the literature, the retrospective nature of the study can be considered a limitation. Second, we did not evaluate the effect of 5-ARI on the clinically significant cancer because of the low number of patients in the subgroups.

In conclusion, our findings suggest that taking 5-ARI increases the sensitivity of mpMRI in detecting cancer but decreases its specificity. In addition, the malignancy rate in PIRADS 4 was higher in patients using 5-ARI. In light of the present findings, urologists and radiologists should be more cautious when interpreting the MRI findings in patients using 5-ARI.

## References

[1] Siegel RL Miller KD Jemal A Cancer statistics, 2018. CA Cancer J Clin 2018 68 7 30 10.3322/caac.21442 29313949

[2] Ketelsen D Röthke M Aschoff P Merseburger AS Lichy MP Reimold M Nachweis ossärer Metastasen des Prostatakarzinoms - Vergleich der Leistungsfähigkeit der Ganzkörper-MRT und der Skelettszintigrafie [Detection of bone metastasis of prostate cancer - comparison of whole-body MRI and bone scintigraphy] RoFo Fortschr Geb Rontgenstr Nuklearmed 2008 180 746 52 10.1055/s-2008-1027479 18512192

[3] Weinreb JC Barentsz JO Choyke PLCornud F, Haider MA, Macura KJ, et alPI-RADS Prostate Imaging - Reporting and Data System: 2015, Version 2. Eur Urol 2016 69 16 40 10.1016/j.eururo.2015.08.052 26427566 PMC6467207

[4] Metens T Miranda D Absil J Matos C What is the optimal b value in diffusion-weighted MR imaging to depict prostate cancer at 3T? Eur Radiol 2012 22 703 9 10.1007/s00330-011-2298-9 21971824

[5] Edwards JE Moore RA Finasteride in the treatment of clinical benign prostatic hyperplasia: a systematic review of randomised trials. BMC Urol 2002 2 14 10.1186/1471-2490-2-14 12477383 PMC140032

[6] Thompson IM Goodman PJ Tangen CM Lucia MS Miller GJ Ford LG The influence of finasteride on the development of prostate cancer. N Engl J Med 2003 349 215 24 10.1056/NEJMoa030660 12824459

[7] Andriole GL Bostwick DG Brawley OW Gomella LG Marberger M Montorsi F Effect of dutasteride on the risk of prostate cancer. N Engl J Med 2010 362 1192 202 10.1056/NEJMoa0908127 20357281

[8] Jacobsen SJ Girman CJ Lieber MM Natural history of benign prostatic hyperplasia. Urology 2001 58 5 16 10.1016/S0090-4295(01)01298-5 11750242

[9] Van Poppel H Hogenhout R Albers P van den Bergh RCN Barentsz JO Roobol MJ A European model for an organised risk-stratified early detection programme for prostate cancer. Eur Urol Oncol 2021 4 731 9 10.1016/j.euo.2021.06.006 34364829

[10] Mottet N, van den Bergh RC, Briers E, Van den Broeck T, Cumberbatch MG, De Santis M, et al. EAU-EANM-ESTRO-ESUR-SIOG guidelines on prostate cancer—2020 update. Part 1: screening, diagnosis, and local treatment with curative intent. 2021;79(2):243-62. 10.1016/j.eururo.2020.09.042 33172724

[11] Nickel JC Comparison of clinical trials with finasteride and dutasteride. Rev Urol 2004 6 Suppl 9 S31 16985923 PMC1472914

[12] Andriole GL Kirby R Safety and tolerability of the dual 5alpha-reductase inhibitor dutasteride in the treatment of benign prostatic hyperplasia. Eur Urol 2003 44 82 8 10.1016/S0302-2838(03)00198-2 12814679

[13] Andriole GL Bostwick D Brawley OW Gomella L Marberger M Montorsi F The effect of dutasteride on the usefulness of prostate specific antigen for the diagnosis of high grade and clinically relevant prostate cancer in men with a previous negative biopsy: results from the REDUCE study. J Urol 2011 185 126 31 10.1016/j.juro.2010.09.011 21074214

[14] Moore CM Robertson NL Jichi F Damola A Ambler G Giganti F The effect of dutasteride on magnetic resonance imaging defined prostate cancer: MAPPED-A randomized, placebo controlled, double-blind clinical trial. J Urol 2017 197 1006 13 10.1016/j.juro.2016.11.090 27871928

[15] Giganti F Moore CM Robertson NL McCartan N Jameson C Bott SRJ MRI findings in men on active surveillance for prostate cancer: does dutasteride make MRI visible lesions less conspicuous? Results from a placebo-controlled, randomised clinical trial. Eur Radiol 2017 27 4767 74 10.1007/s00330-017-4858-0 28523355 PMC5635085

[16] Epstein JI Egevad L Amin MB Delahunt B Srigley JR Humphrey PA Grading Committee. The 2014 International Society of Urological Pathology (ISUP) Consensus Conference on Gleason Grading of Prostatic Carcinoma: Definition of Grading Patterns and Proposal for a New Grading System. Am J Surg Pathol 2016 40 244 10.1097/PAS.0000000000000530 26492179

[17] Iczkowski KA Qiu J Qian J Somerville MC Rittmaster RS Andriole GL The dual 5-alpha-reductase inhibitor dutasteride induces atrophic changes and decreases relative cancer volume in human prostate. Urology 2005 65 76 82 10.1016/j.urology.2004.08.042 15667867

[18] Giganti F Gambarota G Moore CMbertson NL, McCartan N, Jameson C, et alProstate cancer detection using quantitative T_2_ and T_2_ -weighted imaging: The effects of 5-alpha-reductase inhibitors in men on active surveillance. J Magn Reson Imaging 2018 47 1646 53 10.1002/jmri.25891 29135073

[19] Starobinets O Kurhanewicz J Noworolski SM Improved multiparametric MRI discrimination between low-risk prostate cancer and benign tissues in a small cohort of 5alpha-reductase inhibitor treated individuals as compared with an untreated cohort. NMR Biomed 2017 30 10.1002/nbm.3696 28164396 PMC5522750

[20] Bekci T Oguz U Tosun A Demirelli E Ogreden E Sengul D Diagnostic performance of prostate imaging reporting and data system v2.1: Single center experience. Annals of Medical Research 2021 26 1279 82

[21] Loggitsi D Gyftopoulos A Economopoulos N Apostolaki A Kalogeropoulos T Thanos A Multiparametric magnetic resonance imaging of the prostate for tumour detection and local staging: imaging in 1.5t and histopathologic correlation. Can Assoc Radiol J 2017 68 379 86 10.1016/j.carj.2017.02.003 28720413

[22] Woo S Suh CH Kim SY Cho JY Kim SH Diagnostic performance of Prostate Imaging Reporting and data system version 2 for detection of prostate cancer: a systematic review and diagnostic meta-analysis. Eur Urol 2017 72 177 88 10.1016/j.eururo.2017.01.042 28196723

[23] Pokorny MR de Rooij M Duncan E Schröder FH Parkinson R Barentsz JO Prospective study of diagnostic accuracy comparing prostate cancer detection by transrectal ultrasound-guided biopsy versus magnetic resonance (MR) imaging with subsequent MR-guided biopsy in men without previous prostate biopsies. Eur Urol 2014 66 22 9 10.1016/j.eururo.2014.03.002 24666839

[24] Forte V Cavallo AU Bertolo R de Soccio V Sperandio M Bove P PI-RADS score v.2 in predicting malignancy in patients undergoing 5α-reductase inhibitor therapy. Prostate Cancer Prostatic Dis 2021 24 150 5 10.1038/s41391-020-0256-9 32681155

[25] Kim JK Lee HJ Hwang SI Choe G Kim HJ Hong SK The effect of 5 alpha-reductase inhibitor therapy on prostate cancer detection in the era of multi-parametric magnetic resonance imaging. Sci Rep 2019 9 17862 10.1038/s41598-019-54464-9 31780771 PMC6882845

[26] Wang Z Wang K Ong HY Tsang WC Wu QH Chiong E 5-alpha reductase inhibitors and MRI prostates: actively reducing prostate sizes and ambiguity. BMC Urol 2023 23 61 10.1186/s12894-023-01235-4 37061671 PMC10105450

[27] Falagario UG Lantz A Jambor I Busetto GM Bettocchi C Finati M Diagnosis of prostate cancer with magnetic resonance imaging in men treated with 5-alpha-reductase inhibitors. World J Urol 2023 41 2967 74 10.1007/s00345-023-04634-2 37787941 PMC10632288

